# Association between IL-18 -607 C/A Polymorphism and the Risk of Prostate Cancer: A Meta-Analysis of Case-Control Studies

**DOI:** 10.31557/APJCP.2019.20.6.1595

**Published:** 2019

**Authors:** Gao Yuanyuan, Yu Xue, Li Yachao, Feng Xiao, Chen Xu

**Affiliations:** 1 *Department of Clinical Laboratory, First Affiliated Hospital of Soochow University,*; 2 *Central Laboratory of Pediatric Research Institute, Affiliated Children’s Hospital of Soochow University, Suzhou, China.*

**Keywords:** IL-18, prostate cancer, polymorphism, meta-analysis

## Abstract

**Background:**

Accumulating evidence shows that cytokines play an important role in the proliferation of prostate cancer. This research is trying to determine that IL-18 -607 C/A polymorphism confers susceptibility to prostate cancer.

**Methods:**

Meta-analysis was used to collect data. The relevant studies were identified through a comprehensive search from PubMed, Excerpta Medica Database (EMBASE), Web of Science, and Chinese Biomedical Literature Database (CBM) to obtain related studies published up to December 6, 2017. The association between interleukin (IL)-18 -607 C/A polymorphism and prostate cancer risk was assessed by odds ratios (ORs) together with their 95% confidence intervals (CIs).

**Results:**

Nine case-control studies from 6 articles were eventually identified. In the overall population, there is a significant association between IL-18 -607 C/A polymorphism and prostate cancer risk in recessive (CC versus CA/AA: OR = 0.20, 95% CI = 0.15-0.27, P = 0.000) or dominant (CC/CA versus AA:OR = 0.42, 95% CI = 0.30–0.57, P = 0.000) models. In the sub-group analysis according to ethnicity, for Asians, IL-18 -607 C/A polymorphism was significantly associated with prostate cancer in allele contrast (C versus. A: OR=0.82, 95%CI=0.70-0.97, P=0.019), homozygote (CC versus. AA: OR=0.68, 95%CI=0.50-0.92, P=0.013), recessive (CC versus. CA/AA: OR=0.19, 95%CI=0.13-0.27, P=0.000), and dominant (CC/CA versus. AA: OR=0.37, 95%CI=0.28-0.48, P=0.000) models, for Caucasians, IL-18 -607 C/A polymorphism was significantly associated with prostate cancer risk in allele contrast (C versus. A: OR=1.27, 95%CI=1.02-1.58, P=0.033), homozygote (CC versus. AA: OR=1.86, 95%CI=1.19-2.91, P=0.007) and recessive (CC versus. CA/AA: OR=0.25, 95%CI=0.19-0.33, P=0.000) models.

**Conclusion:**

This meta-analysis has shown that IL-18 -607 C/A polymorphism contributes to a decreased risk of prostate cancer risk in the Asian population but an increased risk in the Caucasian population.

## Introduction

Prostate cancer refers to a kind of epithelial malignant tumor that occurs in the prostate of men. In Europe, it is recognized as the most common solid tumor, with an incidence rate of 214 cases per thousand men (Boyle and Ferlay, 2005). Although epidemiological studies have shown that the incidence of prostate cancer in the Asian population is far lower than that in the United States and European countries, the incidence and mortality rate of this disease have increased rapidly in China (Ostrander and Stanford, 2000; Quinn and Babb, 2002). However, the etiology of prostate cancer remains unclear. 

Studies have revealed several risk factors during recent decades, which been associated with increased risk of prostate cancer (Sequoia et al., 2006). Some people develop the disease while others do not, even when everyone is exposed to the same environment, suggesting that gene polymorphism, such as single-nucleotide polymorphism (SNPs) may contribute to prostate cancer carcinogenesis.

Interleukin-18 (IL-18) is a multifunctional cytokine that can induce tumor necrosis factor-α (TNF-α) and interferon-gamma (IFN-g), enhance the cytotoxicity of natural killer (NK) cells and up-regulate the Fas ligand (FasL) expression (Hodge et al., 2006). There is no doubt that IL-18 plays an important role in antitumor immunity. In addition, IL-18 is an critical genetic factor in the proliferation of prostate cancer. Variations in the DNA sequence of IL-18 gene promoter may influence the production and/or activity of IL-18, which modulates an individual’s susceptibility of prostate cancer. IL-18 expression in prostate cancer is also related to the pathologic stage and the transition from androgen-responsive to hormone-refractory of prostate cancer.

The IL-18 gene is located on chromosome 11q22.2-q22.3. Cloning and gene expression analysis suggested that two SNPs of the promoter of IL-18 gene at position -607 (rs 1946518) and -137 (rs 187238) may cause differences in transcription factor binding and have an impact on IL-18 gene activity (Giedraitis et al., 2001). The important polymorphism of IL-18, the C/A mutation at the -607 loci, exists in a variety of tumors, has been particularly considered. In addition, the relationship between IL-18 -607 C/A (rs1946518) polymorphism and prostate cancer has been investigated in previous studies. Although, most of the studies showed that there was no significant association of IL-18 -607 C/A polymorphism in the proliferation of prostate cancer in the Asian population, the result still requires further confirmation. Therefore, a meta-analysis of all eligible case-control studies was performed in order to verify the association between IL-18 -607 C/A polymorphism and prostate cancer risk in this article. To the best of our knowledge, this is the first meta-analysis to discuss the relationship between IL-18 -607 C/A and prostate cancer risk.

## Materials and Methods

*Search strategy*

A comprehensive search was conducted on the electronic databases PubMed, Excerpta Medica Database (EMBASE), Web of Science, and Chinese Biomedical Literature Database (CBM) to obtain related studies published up to December 6, 2017. The method of collecting data is meta-analysis. The search strategy was based on combinations using the following terms: “IL-18” or “Interleukin-18” in combination with “polymorphism” or “variant” or “SNP” or “mutation” in combination with “prostate cancer” or “prostate carcinoma” for all publications on the association between IL-18 -607 C/A polymorphism and prostate cancer risk. No language or country restriction was applied. Other potentially eligible articles were collected by searching from the reference lists of relevant literature and reviews.

*Inclusion and exclusion criteria*

All eligible studies were collected in the meta-analysis according the following inclusive criteria: (a) independent case-control or cohort studies; (b) possessing IL-18 -607C/A (rs 1946518) polymorphism; (c) the paper must provide sufficient genotype data of both cases and controls with odds ratio (OR) and 95% confidence interval (95% CI); (d) if multiple publications from the same or overlapping population were available, the most informative one was included. Studies were excluded if one of the following existed: (a) not case-control studies; (b) not offering the source of cases and controls or other essential information; (c) studies that contained overlapping data; (d) reviews and repeated literature were also excluded.

*Data extraction*

All available data from the eligible studies were extracted according to the standard protocols by two investigators independently. A third investigator joined in the decision-making process if there were any disagreements. The standard protocols are as follows: first author’s name, publication year, ethnicity of the population, country of origin, source of control, the number of cases and controls, genotyping methods, allele frequency, genotype distribution both in case and controls, and the results of the Hardy-Weinberg equilibrium (HWE) test. Different ethnicities of the population were categorized as Caucasian and Asian. 

*Statistical analysis*

STATA software (version 9.0, Stata Crop) was used to analyze the data. The possible association between IL-18 -607 C/A polymorphism and prostate cancer risk was evaluated by odds ratio (OR) and 95% confidence interval (95% CI) in the allele contrast (C versus. A), homozygote (CC versus. AA), heterozygote (CA versus. AA), recessive (CC versus. CA/AA), and dominant (CC/CA versus. AA) models. The heterogeneity between the studies was assessed by the chi-squared (χ^2^)-test based on Q-statistic and I^2^. If the heterogeneity is acceptable (P>0.10 or I^2^ < 50% suggested no obvious heterogeneity), the fixed effect model (the Mantel-Haenszel method (Mantel and Haenszel, 1959)) will be adopted; otherwise, the random effect model (the DerSimonian and Laird method(R and N, 1986)) will be used to calculate the pooled ORs. The random effect model is a method for disposing heterogeneous data, but it cannot replace the analysis of the source of heterogeneity. Several factors might induce the heterogeneity, including design scheme, measuring method, age, and ethnicity. 

Funnel plot was used to observe the possible publication bias in the study visually, and Begg’s and Egger’s tests were used to detect the publication bias statistically. If P<0.05, the result was considered representative of statistically significant publication bias. 

## Results

*Eligible studies*

**Figure 1:**
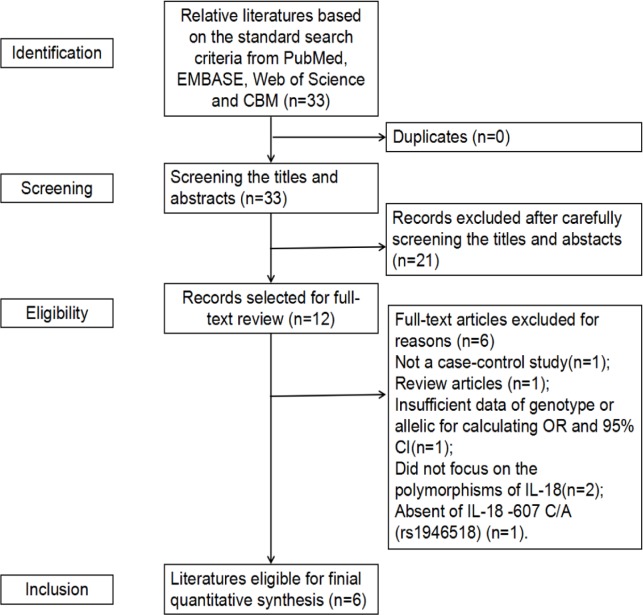
Flow Diagram of Included Studies for This Meta-Analysis

33 relative studies were found based on the standard search criteria. After screening the titles and abstracts, 21 studies were excluded because they were not relevant to the relationship between IL-18 polymorphism and the risk of prostate cancer. Therefore, 12 full-text publications were obtained for further evaluation (Figure 1). According to the exclusion criteria, 6 publications were excluded: 1 publication was not a case-control study (X et al., 2015), 1 was a review article (Veeranki, 2013), 1 for not presenting sufficient genotype or allelic data for calculating OR and 95% CI (Dwivedi et al., 2011), 2 did not focus on the polymorphism of IL-18 (Dwivedi et al., 2012; Shailendra et al., 2015), 1 for absence of IL-18 -607 C/A (rs1946518)(Mi et al., 2017). There was no additional article after manual search of references cited in the published studies. Finally, 6 relative articles (Liu et al., 2007; Liu et al., 2013; Nong et al., 2013; Dwivedi et al., 2015a; Dwivedi et al., 2015b; Jurecekova et al., 2017) were obtained . The search process is shown in Figure 1. Therefore, a total of 6 individual case-control studies, consisting of 1,613 prostate cancer patients and 1,630 controls were included in the meta-analysis ((Dwivedi et al., 2015b) has four separate case-control studies). The selected study characteristics are listed in Table 1. There were 5 case-control studies of subjects of Asian descent (Liu et al., 2007; Liu et al., 2013; Nong et al., 2013; Dwivedi et al., 2015a; Dwivedi et al., 2015b), and 1 case-control study of Caucasians (Jurecekova et al., 2017). The genotype distribution of the control group among all studies were consistent with Hardy-Weinberg equilibrium (HWE) (P >0.05). 

**Figure 2.**
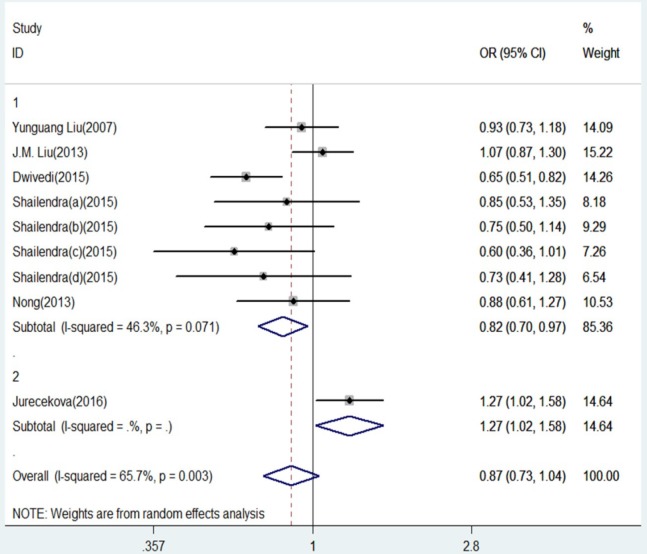
Forest Plot of IL-18 -607 C/A Polymorphism and Prostate Cancer Risk in Allele Contrast (C versus. A). Group 1 represents Asians; Group 2 represents Caucasians

**Figure 3:**
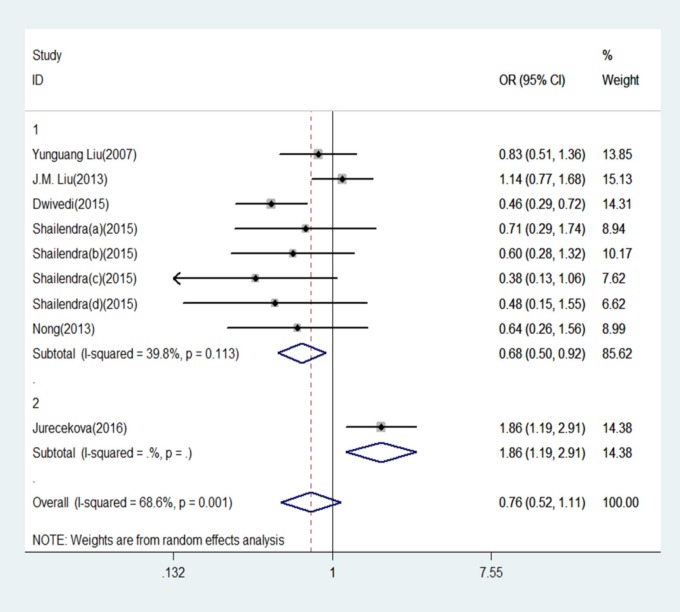
Forest Plot of IL-18 -607 C/A Polymorphism and Prostate Cancer Risk Under Homozygote Model (CC versus. AA).Group 1 represents Asian; Group 2 represents Caucasians

**Figure 4:**
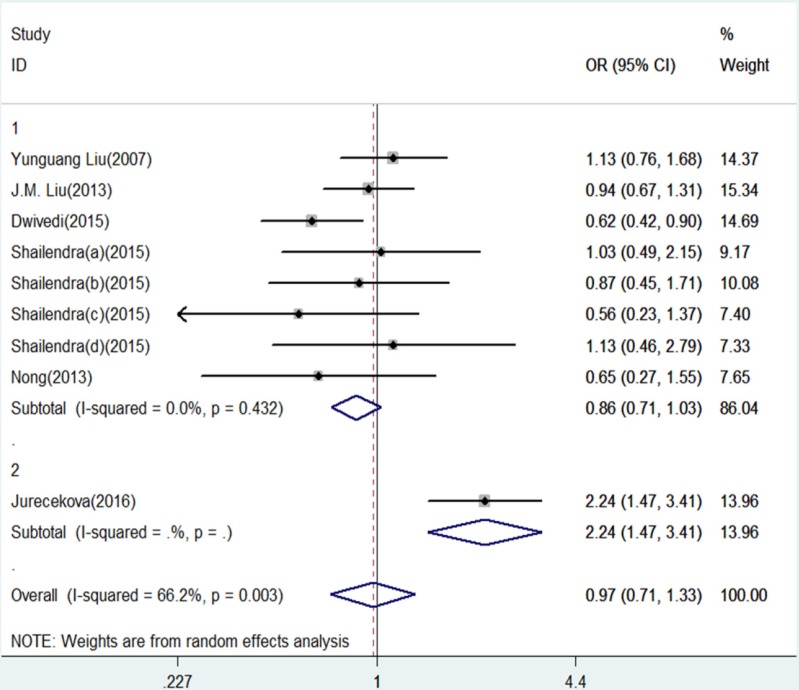
Forest Plot of IL-18 -607 C/A Polymorphism and Prostate Cancer Risk Under Heterozygote Model (CA versus. AA).Group 1 represents Asian; Group 2 represents Caucasians

**Figure 5:**
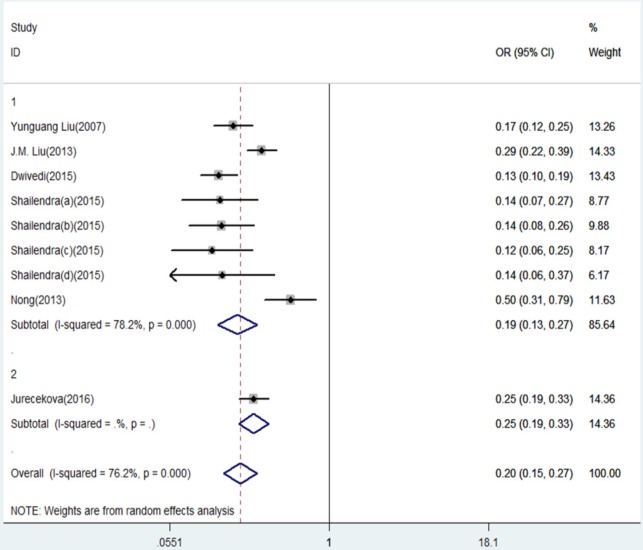
Forest Plot of IL-18 -607 C/A Polymorphism and Prostate Cancer Risk Under Recessive Model (CC versus. CA/AA).Group 1 represents Asian; Group 2 represents Caucasians

**Figure 6:**
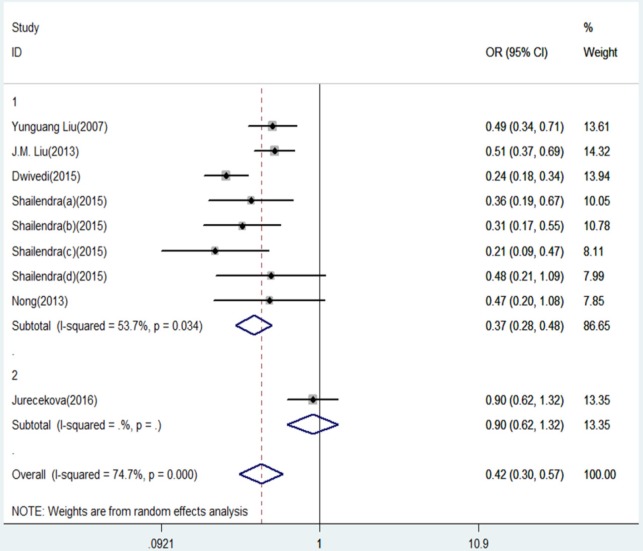
Forest Plot of IL-18 -607 C/A Polymorphism and Prostate Cancer Risk Under Dominant Model (CC/CA versus. AA).Group 1 represents Asians; Group 2 represents Caucasians

**Figure 7:**
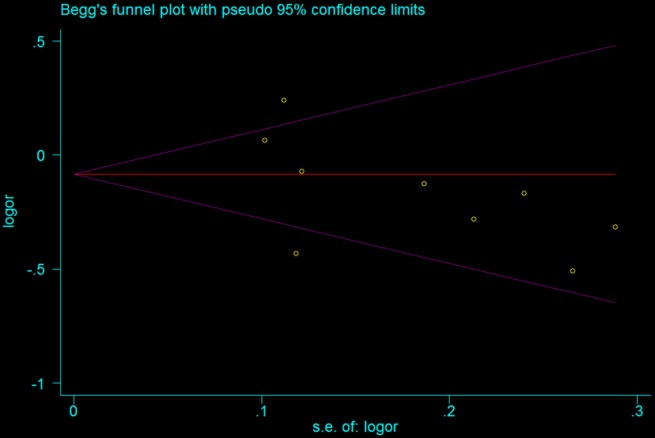
Begg’s Funnel Plot for Publication Bias Test in the Studies of the Meta-Analysis on the Association between IL-18 -607 C/A Polymorphism and Prostate Cancer Risk of the Overall Population (in Allele Contrast C versus. A)

**Table 1:** Characteristics of Eligible Studies

Literature	Country	Ethnicity	Genotyping method	Source of control	Sample size	HWE
Liu (2007)	China	Asian	PCR-SSP	PB	265/280	Yes
Liu (2013)	China	Asian	PCR-SSP	HB	375/400	Yes
Dwivedi (2015)	India	Asian	PCR-RFLP	PB	291/291	Yes
Shailendra (a) (2015)	India	Asian	PCR-RFLP	PB	72/71	Yes
Shailendra (b) (2015)	India	Asian	PCR-RFLP	PB	92/88	Yes
Shailendra (c) (2015)	India	Asian	PCR-RFLP	PB	63/53	Yes
Shailendra (d) (2015)	India	Asian	PCR-RFLP	PB	42/59	Yes
Jurecekova (2016)	Slovak	Caucasians	PCR-SSP	HB	425/263	Yes
Nong (2013)	China	Asian	TaqMan	HB	126/125	Yes

PB, population-based; HB, hospital-based; HWE, Hardy–Weinberg equilibrium in control population; PCR–RFLP, polymerase chain reaction-restriction fragment length polymorphism; PCR-SSP, polymerase chain reaction-sequence-specific primer.

**Table 2:** Results of Meta-Analysis for the IL-18 -607 C/A (rs1946518) and Prostate Cancer Risk

Comparison	Population	N	Test of association			Mode	Test of heterogeneity		
			OR	95%CI	P		χ^2^	P	I^2^
C versus. A	Overall	9	0.87	0.73-1.04	0.13	Rondom	23.31	0.003	65.7
	Asian	8	0.82	0.70-0.97	0.019	Rondom	13.03	0.071	46.3
	Caucasians	1	1.27	1.02-1.58	0.033	/	0.00	/	/
CC versus. AA	Overall	9	0.76	0.52-1.11	0.157	Rondom	25.47	0.001	68.6
	Asian	8	0.68	0.50-0.92	0.013	Rondom	11.63	0.113	39.8
	Caucasians	1	1.86	1.19-2.91	0.007	/	0.00	/	/
CA versus. AA	Overall	9	0.97	0.71-1.33	0.853	Rondom	23.66	0.003	66.2
	Asian	8	0.86	0.71-1.03	0.101	Rondom	6.97	0.432	0.0
	Caucasians	1	2.24	1.47-3.41	0	/	0.00	/	/
CC versus. CA/AA	Overall	9	0.20	0.15-0.27	0	Rondom	33.67	0.000	76.2
	Asian	8	0.19	0.13-0.27	0	Rondom	32.14	0.000	78.2
	Caucasians	1	0.25	0.19-0.33	0	/	0.00	/	/
CC/CA versus. AA	Overall	9	0.42	0.30-0.57	0	Rondom	31.57	0.000	74.7
	Asian	8	0.37	0.28-0.48	0	Rondom	15.12	0.034	53.7
	Caucasians	1	0.90	0.62-1.32	0.598	/	0.00	/	/

*Quantitative synthesis of data*

The results of this meta-analysis for the association between IL-18 -607 C/A (rs1946518) and prostate cancer risk are displayed in Table 2. We found that, in the overall population, there is a significant association between IL-18 -607 C/A polymorphism and prostate cancer risk using a recessive (CC versus CA/AA: OR = 0.20, 95% CI = 0.15-0.27, P = 0.000, Figure 5) or dominant (CC/CA versus AA:OR = 0.42, 95% CI = 0.30–0.57, P = 0.000, Figure 6) model. In the sub-group analysis according to ethnicity, showed in the meta-analysis, for Asians, IL-18 -607 C/A polymorphism was significantly associated with prostate cancer in allele contrast (C versus. A: OR=0.82, 95% CI=0.70-0.97, P=0.019, Figure 2), homozygote (CC versus. AA: OR=0.68, 95% CI=0.50-0.92, P=0.013, Figure 3), recessive (CC versus. CA/AA: OR=0.19, 95% CI=0.13-0.27, P=0.000, Figure 5), and dominant (CC/CA versus. AA: OR=0.37, 95%CI=0.28-0.48, P=0.000, Figure 6) models. For Caucasians, IL-18 -607 C/A polymorphism was significantly associated with prostate cancer risk in allele contrast (C versus. A: OR=1.27, 95% CI=1.02-1.58, P=0.033, Figure 2), homozygote (CC versus. AA: OR=1.86, 95% CI=1.19-2.91, P=0.007, Figure 3) and recessive (CC versus. CA/AA: OR=0.25, 95% CI=0.19-0.33, P=0.000, Figure 5) models. If OR more than 1 means high risk while OR less than 1 lower risk. 

*Sensitivity analysis*

Individual studies were consecutively omitted in the sensitivity analysis to detect the influence of each study on the pooled OR. In the meta-analysis, no single study significantly influenced the overall results as indicated by sensitivity analysis. That means the results of the meta-analysis were robust and reliable.

*Publication bias*

We adopted the Begg’s funnel plot and Egger’s test to evaluate the publication bias of articles in this meta-analysis. As shown in Figure 7, the shapes of the funnel plot were symmetrical, suggesting that there was no evidence of publication bias in allele contrast in this meta-analysis. Therefore, the results are reliable according to the included articles.

## Discussion

Many studies in recent years have indicated that SNPs are the most common sources of human genetic variation, and they are likely to contribute to an individual’s susceptibility to cancer (Wu et al., 2009). Interest in the genetic susceptibility to cancers has resulted in growing attention to the study of polymorphism of genes involved in tumorigenesis. To offset the lack of epidemiological studies of IL-18 -607 C/A polymorphism and prostate cancer risk, this meta-analysis can provide further data on the relationship between this gene and prostate cancer risk.

In this study, we included data from 9 case-control comparisons with more than 3,000 genotyped prostate cancer patients and controls. According to the study design, 3 studies were conducted in a hospital-based design (Liu et al., 2013; Nong et al., 2013; Jurecekova et al., 2017), and 6 in a population-based design (Liu et al., 2007; Dwivedi et al., 2015a; Dwivedi et al., 2015b). The overall data suggested that IL-18 -607 C/A is not associated with the prostate cancer risk. However, the stratified analysis by ethnicity yielded different findings. In the Asian population, although all the articles included in the meta-analysis showed that there is no association between IL-18 -607 C/A and prostate cancer risk, after meta-analysis, we found that IL-18 -607 C/A is associated with the prostate cancer risk as a protective factor, and is also associated with the prostate cancer risk in Caucasians correspondingly. In general, IL-18 -607 C/A polymorphism plays different roles in prostate cancer susceptibility among different ethnic subgroups. Combining data from as many studies as possible has the advantage of reducing random error (Ioannidis et al., 2008).

Heterogeneity may be a potential problem impacting the results of meta-analysis. Significant between-study heterogeneity in the overall analysis existed under almost all the gene models in the study. However, after subgroup analysis by ethnicity, the heterogeneity was removed in almost all of the gene models in the Asian population. There are many factors that could have contributed to the high heterogeneity. Racial differences may be the most important reason for the genetic heterogeneity. As we all know, the relationship between the genotypes distributed in different populations may also depend on the population stratification. Second, the source of control is different in different studies, which may have resulted in heterogeneity in the meta-analysis. Other reasons that may account for the heterogeneity included different genotyping method and publication bias. The currently available data on the association between IL-18 -607 C/A polymorphism and prostate cancer in Caucasians are still very limited, so it should be interpreted with caution. Further studies are required to provide an authentic result.

Some limitations of this meta-analysis should be acknowledged. First, only published studies in English were included in the meta-analysis, so publication biases and English language biases were unavoidable. Second, the results are based on unadjusted estimates, and a more precise analysis stratified by age, different living environment, lifestyle factors, such as smoking and drinking, and different grades of prostate cancer could be performed if individual data were available. Third, the result should be cautiously interpreted because controls were not uniformly defined. These studies may have included controls that had different risks for developing prostate cancer in the future. Fourth, only one study included in this meta-analysis analyzed the Caucasian population. Therefore, more studies of the Caucasian population are needed in the future to obtain more precise conclusions about the associations between IL-18 -607 C/A polymorphism and prostate cancer risk. 

Despite these limitations or disadvantages, the meta-analysis still had some advantages. First, this is the first meta-analysis on the association between IL-18 -607 C/A polymorphism and prostate cancer risk. Second, a systematic review of the association of IL-18 -607 C/A polymorphism and prostate cancer risk is statistically far more powerful than any single study. Third, the sensitivity analysis and publication bias analysis showed the stability and credibility of the meta-analysis, and the process of literature selection, data extraction, and data analysis in the meta-analysis was well designed and conducted.

In conclusion, the present meta-analysis provides information that there is no association between IL-18 -607 C/A polymorphism and prostate cancer risk in the overall population, but in the stratified analysis by ethnicity, IL-18 -607 C/A polymorphism was found to be associated with prostate cancer risk as a protect factor in the Asian population and also associated with prostate cancer risk in Caucasians. Larger scale primary studies with the consideration of gene-gene and gene-environment interactions are required to further evaluate the interaction of IL-18 -607 C/A polymorphism with prostate cancer susceptibility.
